# Association between early mobilization after hip fracture surgery and risk of long-term opioid therapy

**DOI:** 10.1007/s41999-025-01227-7

**Published:** 2025-05-07

**Authors:** Yasmina Maria Tudorache, Ina Trolle Andersen, Thomas J. Hjelholt, Morten Tange Kristensen, Katie J. Sheehan, Alma B. Pedersen

**Affiliations:** 1 Department of Clinical Epidemiology, Department of Clinical Medicine, Aarhus University Hospital, Aarhus University, Olof Palmes Alle 43-45, 8200 Aarhus N, Aarhus, Denmark; 2 Department of Geriatrics, Aarhus University Hospital, Aarhus, Denmark; 3 Department of Physical and Occupational Therapy, Bispebjerg and Frederiksberg and Department of Clinical Medicine, Copenhagen University Hospital, University of Copenhagen, Copenhagen, Denmark; 4 Bone and Joint Health, Blizard Institute, Queen Mary University of London, London, UK

**Keywords:** Early mobilization, Epidemiology, Geriatrics, Hip fracture, Opioid use, Rehabilitation

## Abstract

**Key summary points:**

**Aim:**

To examine the association between early mobilization after hip fracture surgery and the risk of long-term opioid therapy at 1-year follow-up.

**Findings:**

Long-term opioid therapy is a common complication after hip fracture surgery.

Mobilization within 24 h after surgery is associated with a lower risk of long-term opioid therapy compared to mobilization between 24 and 36 h.

**Message:**

Early mobilization is one of the key elements of the successful patient recovery for reducing risk of complications and mortality after hip fracture surgery.

**Abstract:**

**Purpose:**

Early mobilization after hip fracture operation is associated with better clinical outcomes, but its impact on long-term opioid therapy (LTOT) remains unclear.

**Methods:**

Using Danish population-based registries we included patients aged ≥ 65 who underwent surgery for a first-time hip fracture between 2016 and 2021 (*n* = 36,229). LTOT was defined as redeeming ≥ 2 prescriptions between 31 and 365 days of surgery. Using stabilized inverse probability of treatment (sIPT) weighing, we calculated risks and risk differences with 95% confidence intervals (CI) for opioid use balancing mobilization groups ≤ 24 h vs 24–36 h on measured confounders and taking death into consideration.

**Results:**

67.3% of all patients were women and the median age was 82.6 years (75.8; 88.6). 75% of patients were mobilized ≤ 24 h of surgery, whereas 8% were mobilized between 24 and 36 h, 4.9% > 36 h, and 12.1% had missing data on mobilization. Patients mobilized ≤ 24 h and 24–36 h were similar in age, fracture type, and marital status, but those mobilized ≤ 24 h had fewer comorbidities, better pre-fracture mobility, and a higher social position. They also had a lower risk of LTOT (29.99%) compared to those mobilized 24–36 h (33.42%), with a weighted risk difference of 3.44% (95% CI 1.58–5.30).

**Conclusions:**

LTOT is common after hip fracture surgery. Mobilization ≤ 24 h after surgery is associated with a lower risk of LTOT compared to mobilization between 24 and 36 h. Early mobilization is one of the key elements of successful patient recovery for reducing complications and mortality after hip fracture surgery.

**Supplementary Information:**

The online version contains supplementary material available at 10.1007/s41999-025-01227-7.

## Introduction

Hip fractures are a major public health problem globally [1] with incidence expected to increase due to population aging [2]. Hip fracture is associated with a 1-year mortality up to 30%, which is influenced by patient case mix, postoperative complications, and system factors [3–6].

To mitigate these poor outcomes, early surgery and mobilisation, orthogeriatric care, and rehabilitation are recommended. Opioids are used for managing acute pain after hip fracture surgery. Prescription of opioids beyond the acute phase and up to 3 months after hip fracture has increased [7] with up to 17% of opioid-naive patients $\ge$ 65 years still using opioids 1 year after fracture [8]. This is problematic as older patients are at increased risk of experiencing adverse effects from opioids [9] due to age-related physiological alterations and polypharmacy [10].

Early mobilization within the first one or two postoperative days after hip fracture is associated with lower rates of postoperative complications, recovery of pre-fracture mobility, and lower mortality [11–16]. The relationship between mobilization time and postoperative pain and opioid use after hip fracture is unknown. There is some evidence to indicate early mobilization was associated with shorter duration and intensity of postoperative pain [17], and early physical therapy associated with a 10% reduction in long-term opioid use among those with musculoskeletal pain [18].

Therefore, the aim of this study was to examine the association between early mobilization after hip fracture surgery and the risk of long-term opioid therapy at 1-year follow-up.

## Methods

### Design

This is a population-based cohort study in a Danish setting with free and universal access to tax-financed healthcare for all Danish citizens. The study follows the REporting of studies Conducted Using Observational Routinely‐collected Data (RECORD) guidelines.

### Data sources

We used four Danish population-based medical databases in this study, the Danish Multidisciplinary Hip Fracture Registry (DMHFR [19], the Danish Civil Registration System, the Danish National Patient Registry (DNPR), the Danish National Prescription Registry [20], and Statistics Denmark [21]. Diagnoses are coded after the International Classification of Diseases 10 th edition, while operations are coded after the Danish version of the Nordic Medico-Statistical Committee Classification of Surgical Procedures [22]. The unique 10-digit civil personal registration (CPR) number assigned to each Danish citizen at birth or upon immigration allows for valid crosslinking of data in between different databases on an individual level.

### Study population

The DMHFR was used to identify the study population including patients aged 65 years or older who had sustained a hip fracture (including femoral neck, per- or subtrochanteric fracture) and were treated with the operative procedure of internal fixation or total/hemi arthroplasty. Diagnoses and operation codes are listed in Supplementary information S1 Table 1.

**Table 1:** Characteristics of the hip fracture study population by mobilization status

	Mobilization < = 24 h	Mobilization 24–36 h	Mobilization > 36 h	Missing mobilization
Number of patients, N (%)	27,174 (75.0)	2,890 (8.0)	1,764 (4.9)	4,401 (12.1)
Female, N (%)	18,369 (67.6)	2,001 (69.2)	1,161 (65.8)	2,864 (65.1)
Age (y) at surgery date, median (Q1–Q3)	82.5 (75.7;88.4)	82.9 (76.0;88.7)	83.6 (77.1;89.7)	82.8 (76.0;88.7)
Age (y), N (%)				
65–69	2,434 (9.0)	239 (8.3)	130 (7.4)	397 (9.0)
70–74	3,769 (13.9)	393 (13.6)	217 (12.3)	555 (12.6)
75–79	4,631 (17.0)	489 (16.9)	261 (14.8)	755 (17.2)
80–84	5,613 (20.7)	581 (20.1)	370 (21.0)	912 (20.7)
> = 85	10,727 (39.5)	1,188 (41.1)	786 (44.6)	1,782 (40.5)
Surgery year, N (%)				
2016	4,363 (16.1)	477 (16.5)	348 (19.7)	1,082 (24.6)
2017	4,260 (15.7)	654 (22.6)	306 (17.3)	862 (19.6)
2018	4,218 (15.5)	530 (18.3)	276 (15.6)	981 (22.3)
2019	4,599 (16.9)	464 (16.1)	227 (12.9)	627 (14.2)
2020	4,887 (18.0)	412 (14.3)	304 (17.2)	494 (11.2)
2021	4,847 (17.8)	353 (12.2)	303 (17.2)	355 (8.1)
Region				
Missing information	31 (0.1)	< 5	< 5	< 65(1.5)
Capital Region of Denmark	5,588 (20.6)	1,186 (41.0)	458 (26.0)	2,394 (54.4)
Region Zealand	4,351 (16.0)	543 (18.8)	512 (29.0)	530 (12.0)
Region of Southern Denmark	7,171 (26.4)	559 (19.3)	302 (17.1)	625 (14.2)
Central Denmark Region	6,597 (24.3)	386 (13.4)	332 (18.8)	461 (10.5)
North Denmark Region	3,436 (12.6)	< 220 (7.6)	< 160 (9.1)	< 335 (7.6)
Type of fracture, N (%)				
Femoral neck	15,624 (57.5)	1,684 (58.3)	935 (53.0)	2,487 (56.5)
Per and Sub trochanteric	11,550 (42.5)	1,206 (41.7)	829 (47.0)	1,914 (43.5)
Type of surgery, N (%)				
Osteosynthesis	17,141 (63.1)	1,719 (59.5)	1,113 (63.1)	2,783 (63.2)
Total and hemi hip arthroplasty	10,033 (36.9)	1,171 (40.5)	651 (36.9)	1,618 (36.8)
Surgery delay (h), median (Q1–Q3)	18.5 (10.8;24.6)	19.9 (14.7;28.2)	20.2 (12.5;28.0)	21.2 (14.8;31.3)
Surgery delay (h), N (%)				
Missing	192 (0.7)	15 (0.5)	27 (1.5)	43 (1.0)
< = 12	7,522 (27.7)	420 (14.5)	390 (22.1)	769 (17.5)
> 12–18	5,347 (19.7)	733 (25.4)	327 (18.5)	792 (18.0)
> 18–24	6,826 (25.1)	822 (28.4)	403 (22.8)	1,074 (24.4)
> 24	7,287 (26.8)	900 (31.1)	617 (35.0)	1,723 (39.2)
Living situation, N (%)				
Own residence	20,511 (75.5)	1,923 (66.5)	1,200 (68.0)	2,456 (55.8)
Residential institution, e.g., nursing home	5,352 (19.7)	607 (21.0)	461 (26.1)	848 (19.3)
Other	1,311 (4.8)	360 (12.5)	103 (5.8)	1,097 (24.9)
Marrital status, N (%)				
Unmarried	2,114 (7.8)	227 (7.9)	145 (8.2)	375 (8.5)
Married	9,360 (34.4)	931 (32.2)	536 (30.4)	1,359 (30.9)
Divorced	3,646 (13.4)	421 (14.6)	254 (14.4)	721 (16.4)
Widowed	12,054 (44.4)	1,311 (45.4)	829 (47.0)	1,946 (44.2)
Education level, N (%)				
High	3,550 (13.1)	417 (14.4)	199 (11.3)	557 (12.7)
Medium	9,242 (34.0)	1,049 (36.3)	580 (32.9)	1,501 (34.1)
Low	13,149 (48.4)	1,268 (43.9)	885 (50.2)	2,068 (47.0)
Missing	1,233 (4.5)	156 (5.4)	100 (5.7)	275 (6.2)
BMI, median (Q1–Q3)	23.5 (21.0;26.0)	23.0 (21.0;26.0)	23.1 (21.0;26.3)	23.0 (21.0;26.0)
BMI, N (%)				
Missing	3,693 (13.6)	792 (27.4)	368 (20.9)	1,499 (34.1)
Underweight	2,264 (8.3)	239 (8.3)	136 (7.7)	303 (6.9)
Normal	12,265 (45.1)	1,088 (37.6)	719 (40.8)	1,468 (33.4)
Overweight	6,648 (24.5)	553 (19.1)	382 (21.7)	850 (19.3)
Obese	2,304 (8.5)	218 (7.5)	159 (9.0)	281 (6.4)
CAS pre-fracture, N (%)				
CAS Missing	1,159 (4.3)	234 (8.1)	199 (11.3)	1,136 (25.8)
CAS 0–4	2,800 (10.3)	362 (12.5)	293 (16.6)	568 (12.9)
CAS 5–6	23,215 (85.4)	2,294 (79.4)	1,272 (72.1)	2,697 (61.3)
Number of comorbidities, N (%)				
0	6,031 (22.2)	534 (18.5)	258 (14.6)	768 (17.5)
1–2	9,969 (36.7)	1,029 (35.6)	571 (32.4)	1,464 (33.3)
3–4	6,590 (24.3)	753 (26.1)	479 (27.2)	1,151 (26.2)
+ 5	4,584 (16.9)	574 (19.9)	456 (25.9)	1,018 (23.1)
Opioid use 30 days before surgery, N (%)	3,617 (13.3)	427 (14.8)	285 (16.2)	697 (15.8)
Death after surgery, but before follow-up at day 2, N (%)	68 (0.3)	5 (0.2)	< 5	177 (4.0)

The numbers in the Median (IQR) cells stand for the 25 th and 75 th percentile values

The numbers in the parentheses next to the absolute number—N(%)—denote percentages representing the proportion of a given number relative to the total patient population within its corresponding mobilization subgroup. *BMI* body mass index, *CAS* cumulated ambulation score, *IQR* interquartile range, *N* number, *y* years; *h* hours

**Fig. 1:**
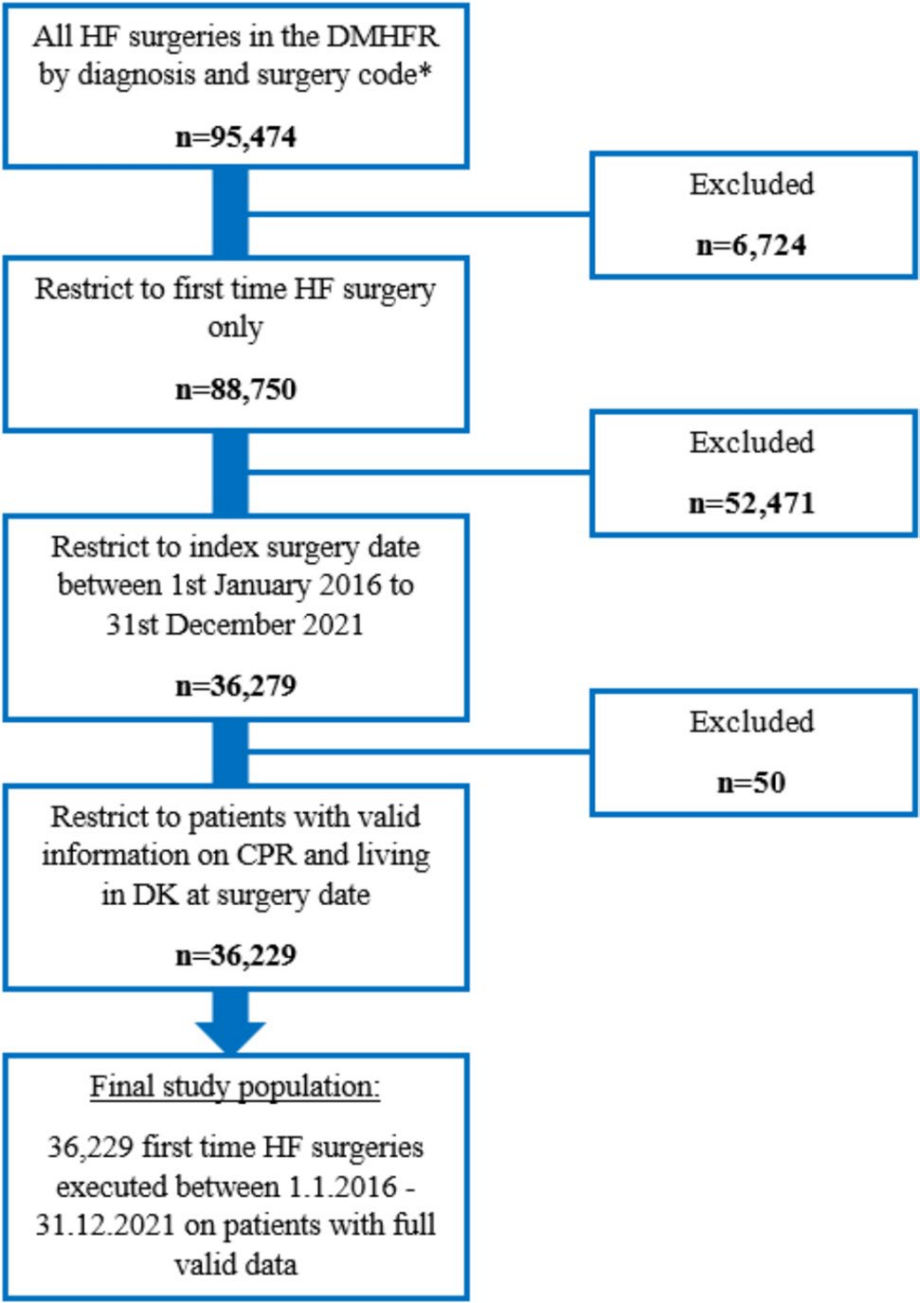
Flow diagram of the hip fracture (HF) study population. The numbers (n = …) denote the absolute number of patients that were excluded at each step of restriction (right side) and the remaining patients after every restriction step (left side). *Femoral neck, pertrochanteric, or subtrochanteric hip fracture diagnosis codes DS72.0-DS72.2 and hip osteosynthesis or total/hemi arthroplasty surgery codes KNFB.0–99 or KNFJ.4–9

We identified 95,474 hip fracture operations in the DMHFR. We included first (index) hip fracture surgery corresponding to 88,750 patients. Patients with periprosthetic or pathological hip fracture were not included per definition in the DMHFR. Since registration of date-time stamp of mobilization became mandatory at the end of 2015, we further restricted the population to surgeries performed between January 1 st 2016 and December 31 st 2021, resulting in 36,279 patients. Finally, we restricted to patients with valid information about the CPR number and living in Denmark at time of surgery resulting in the final study population of 36,229 patients (Fig. 1).

### Early mobilization

Early mobilization was defined in the DMHFR as mobilization within 24 h after surgery knife-start [23]. The exact date, hour and minute of surgery start and first-time mobilization after surgery were recorded in the DMHFR. Using this information, we calculated the time from surgery start to first mobilization in hours. The mobilization time was then dichotomized into ≤ 24 h and 24–36 h for the primary analysis. Secondary analyses used two subgroupings: a) ≤ 24, 24–36, > 36 h, or missing; and b) 5 subgroups based on 12-h intervals: ≤ 12, > 12–24, > 24–36, > 36 h, or missing.

### Opioid use

The outcome of interest was long-term opioid therapy, defined as redeeming 2 or more opioid prescriptions from a community pharmacy from day 31 and up to 1 year after surgery date. Prescription of opioids up to 30 days was considered as part of acute treatment and phase-out plan for opioids. Prescription data were collected from the Danish National Prescription Registry using Anatomical Therapeutic Chemical Classification (ATC) codes of 13 common types of opioids as listed in Supplementary information S1 Table 1. Buprenorphine, codeine and paracetamol, fentanyl, hydromorphone, ketobemidone and antispasmodics, methadone, morphine, nicomorphine, oxycodone, oxycodone and naloxone, pethidine, tapentadol and tramadol were the opioids included in this outcome definition.

### Covariates

The following covariates were retrieved as potential confounders:

From the DMHFR: sex (male, female), age (in categories 65–69, 70–74, 75–79, 80–84, and ≥ 85 years), year of surgery (2016–2021), fracture type (femoral neck, per-/subtrochanteric fracture), surgical delay (≤ 12, 12–18, 18–24, and > 24 h), body mass index (BMI) in categories underweight, normal, overweight, obese [24], and pre-fracture mobility defined though the cumulated ambulation score (CAS) (0–4, 5–6) [25].

From the DNPR: count of 27 major comorbidities (with a prevalence of > 1% in a hip fracture population) recorded within 10 years prior to the index date [26].

From Statistics Denmark: highest attained education, residence, marital status, and region of residence. Education was classified into three categories: low (none or elementary school), medium (more than elementary school but less than university education), and high (university education). Residence was classified as living in own residence, nursing home or other residence. Marital status was classified as being married, unmarried, divorced or widowed.

### Statistics

Patient characteristics by early mobilization status were summarized using counts and percentages for categorical variables and medians with interquartile range for continuous variables. We computed crude cumulative incidences (risks) and risk differences using the Aalen–Johanson estimator treating death as a competing event [27].

To balance measured confounders in the primary analysis, we used the stabilized inverse probability of treatment (sIPT) weighting method [28]. The sIPT weights were derived from propensity scores; these propensity scores were computed using logistic regression to predict the probability of being mobilized ≤ 24 h or within 24–36 h as a function of the selected confounders. Weights were then stabilized by multiplying the weights in the group mobilized ≤ 24 h by the proportion of patients mobilized ≤ 24 h in the total study population and by multiplying the weights in the group mobilized within 24–36 h by the proportion of patients mobilized within 24–36 h in the total study population. We then computed sIPT weighted risks and risk differences, comparing patients mobilized within 24 h and those mobilized within 24–36 h. Standardized mean differences were used to assess the balance of confounders in the two groups, with the aim of achieving a standardized mean difference of less than 0.1 for all confounders**.**

We hypothesized that the main reason why patients were mobilized after 36 h was their morbidity which we potentially were not able to capture by measured confounders; therefore, we did not perform sIPT weighting analyses comparing mobilization < 36 vs > 36 h. However, in the secondary analyses, we calculated the crude risks of long-term opioid therapy with death as competing risk for mobilization divided into a) 4 subgroups defined as ≤ 24, 24–36, > 36 h or missing and b) 5 subgroups with 12-h intervals.

For the statistical analyses we used SAS (version 9.4, SAS Institute Inc., Cary, NC).

### Ethical approval and patient consent

The study was reported to the Danish Data Protection Agency through registration at Aarhus University (record number: AU-2016–051-000001, sequential number 880). Patient consent is not required by Danish law for registry-based studies.

## Results

### Description of the study population

Characteristics of the 36,229 patients are presented in Table 1. The patient characteristics that were similar across all mobilization groups (≤ 24 h, 24–36 h, > 36 h, missing) included median age, age distribution, type of fracture, and marital status. Compared to patients mobilized between 24 and 36 h, those mobilized within 24 h had shorter surgery delays, shorter hospital stays, were more likely to live in their own residence, had a higher prevalence of normal BMI, a higher prevalence of pre-fracture CAS of 5–6, fewer comorbidities, and lower levels of education. Patients mobilized > 36 h or those with missing data on mobilization had more comorbidities than those mobilized ≤ 24 h or within 24–36 h (Table 1).

Most patients were mobilized within 24 h of surgery (75%), whereas 8% of patients were mobilized between 24 and 36 h and 12.1% had missing data on time to mobilization. To address the uneven distribution of patients and confounders, sIPT weighting was applied. This approach balanced the distribution of measured confounders across mobilization groups. After weighting, all standardized mean differences were below 0.1, indicating sufficient covariate balance and supporting the comparability of the groups in subsequent analyses. In addition, the proportion of patients with missing information on mobilization status decreased from 2016 to 2021. This trend may reflect the time required—from months to years—for healthcare workers and administrative staff to fully adapt to recording new quality indicators accurately in online databases.

### Primary analysis—early mobilization and long-term opioid therapy

**Fig. 2:**
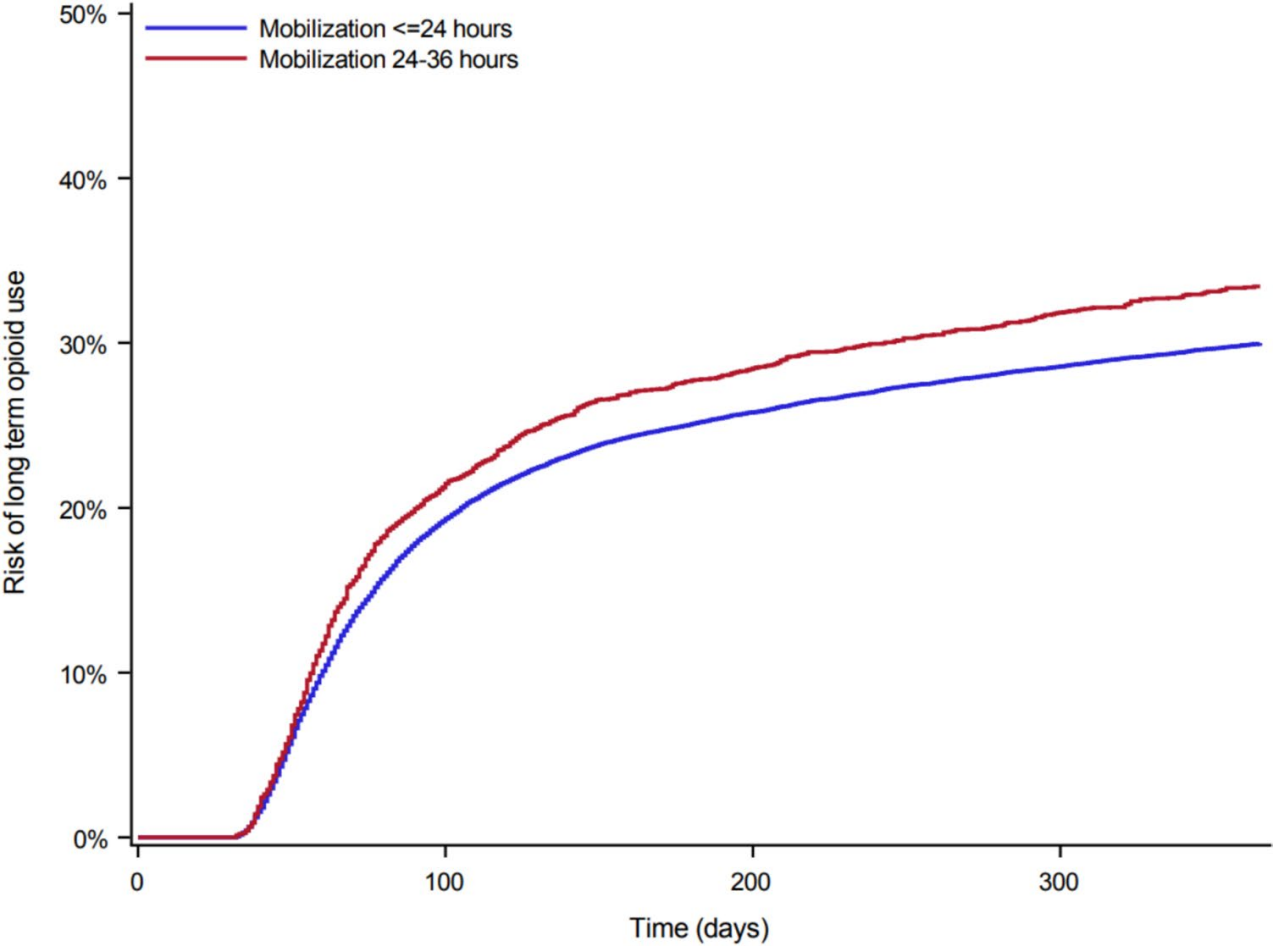
Stabilized inverse probability of treatment weighted cumulative incidences (risks) for long-term opioid therapy within 30 days to 1 year of follow-up by mobilization ≤ 24 and 24–36 h

Confounders were well-balanced after using the sIPT weights. The risks of long-term opioid therapy within 1 year of surgery based on weighted sIPT were 33.42% (95% CI 31.66–35.20) for patients mobilized within 24–36 h and 29.99% (95% CI 29.43–30.55) for patients mobilized ≤ 24 h. This corresponds to a risk difference of 3.44%-points (95% CI 1.58–5.30) for patients mobilized within 24–36 h compared to patients mobilized ≤ 24 h (Table 2**, **Fig. 2**).**

**Table 2:** Main analysis crude and sIPT weighted risk of long-term opioid therapy 1 year after hip fracture surgery by mobilization within 24 h or 24–36 h

Time to mobilization	Number of events	Number at risk	Risk of LTOT (%) (CIF (95% CI))	Risk difference (%) (CIF DIFF (95% CI))
Crude analysis				
< = 24 h	7746	27,106	29.86	Reference
			(29.30,30.42)	
24–36 h	946	2885	33.73	3.88
			(31.98,35.49)	(2.03,5.72)
sIPT weighted analysis				
< = 24 h	7791	27,109	29.99	Reference
			(29.43,30.55)	
24–36 h	920	2871	33.42	3.44
			(31.66,35.20)	(1.58,5.30)

Comparison between the different mobilization groups. The follow-up duration is 1 year for all the included patients. Risks were calculated with death as competing risk. *CI* confidence interval, *CIF* cumulative incidence function, *DIFF* difference, *LTOT* long-term opioid therapy, *sIPT* stabilized inverse probability of treatment

### Secondary analysis—crude risks

**Fig. 3:**
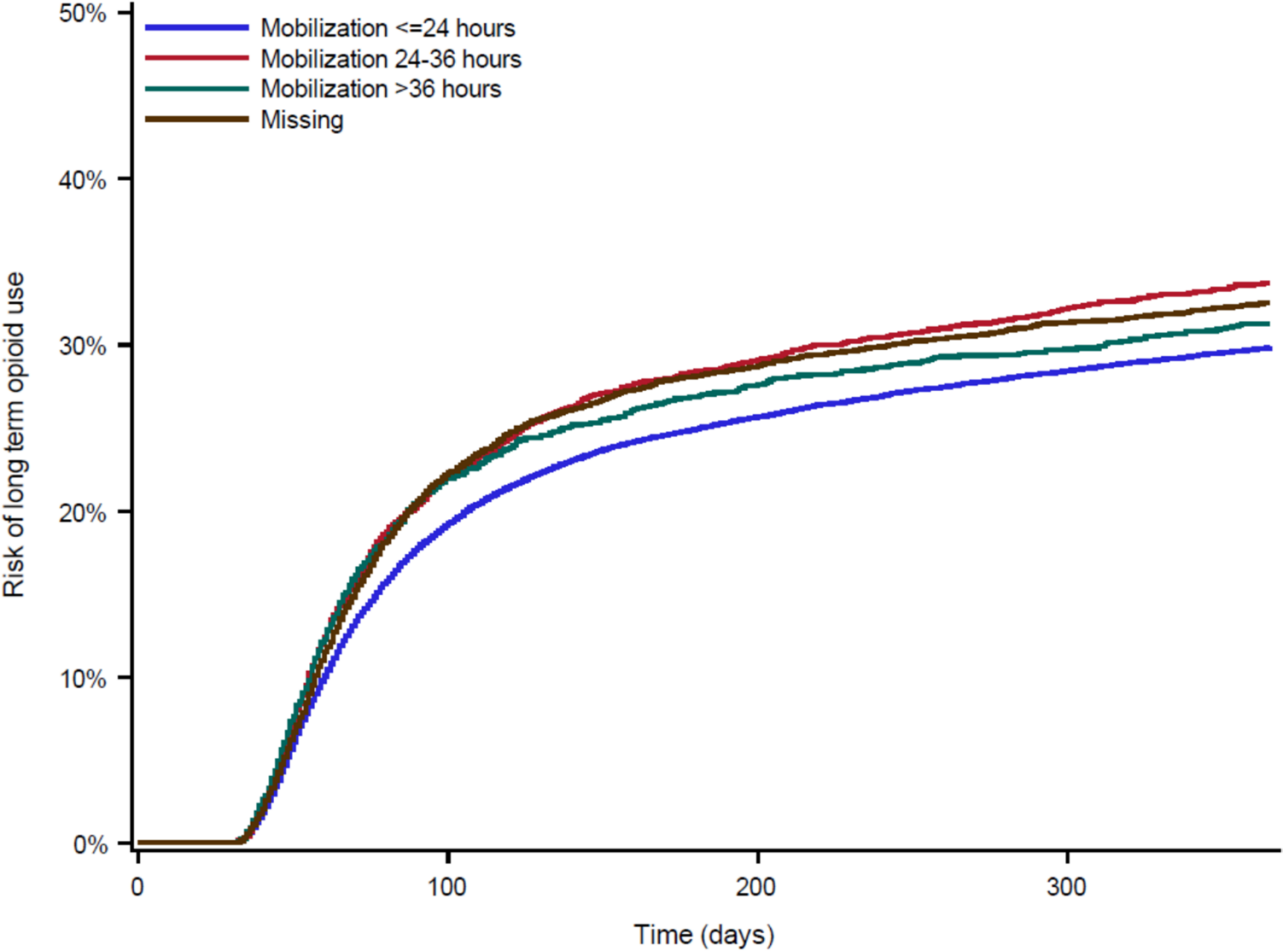
Crude cumulative incidence (risks) for long-term opioid therapy within 30 days to 1 year of follow-up stratified by mobilization subgroup, <  = 24, 24–36, > 36 h or missing data on mobilization

The crude risks of long-term opioid therapy within 1 year of surgery were 29.86%, 33.73%, 31.26% and 32.54% for patients mobilized ≤ 24, 24–36, > 36 h or missing, respectively (Fig. 3**, **Table 3). The group of patients mobilized within 12 or 12–24 h had crude risk of long-term opioid therapy of 27.10% and 30.64%, respectively (Table 3).

**Table 3:** Crude risks of long-term opioid therapy up to 1 year after hip fracture surgery by time to mobilization

Time to mobilization	Risk of LTOT (%) (CIF (95% CI))
< = 12 h	27.10 (25.95,28.26)
> 12–24 h	30.64 (30.01,31.28)
< = 24 h	29.86 (29.30,30.42)
> 24–36 h	33.73 (31.98,35.49)
> 36 h	31.26 (29.07,33.48)
Missing	32.54 (31.12,33.97)

Comparison between the different mobilization groups. The follow-up duration is 1 year for all the included patients. Risks were calculated with death as competing risk. *CI* confidence interval, *CIF* cumulative incidence function, *LTOT* long-term opioid therapy

## Discussion

Mobilization within 24 h was associated with a 3.44%-point lower risk of long-term opioid therapy up to 1 year after hip fracture surgery compared to mobilization within 24–36 h after balancing confounders using the sIPT weighting method. Patients mobilized after 36 h or with missing data on mobilization time did not have additional increased crude risk of long-term opioid therapy compared to patients mobilized within 24–36 h. Patients mobilized within 12 h had the lowest crude risk of long-term opioid therapy.

Most mobilization subgroups follow a dose–response relationship, where earlier mobilization is associated with a lower risk of LTOT. This is shown in the crude risk estimates, which increase from 27.1% among those mobilized within 12 h to 31.26% among those mobilized over 36 h. However, the 24–36-h group deviates from this trend, showing the highest LTOT risk at 33.73%. This difference could potentially be explained by system factors. Patients mobilized over 36 h were more comorbid, had worse pre-fracture mobility and were to a higher extent already institutionalized in nursing homes—factors that may reflect a higher frailty burden, although frailty was not directly measured in this study. Paradoxically, this group had slightly lower opioid use than the 24–36-h group, which may be explained by the higher proportion of nursing home residents in the > 36-h group (26.1%) compared to the 24–36-h group (21.0%). These patients likely had more structured medication management in long-term care facilities by nursing staff, including consistent opioid tapering or discontinuation, potentially lowering the risk of long-term opioid therapy. However, since our sIPT-weighted analysis did not include the > 36-h group due to high risk of unmeasured confounders, further research is needed to confirm whether these differences in long-term opioid therapy would persist after adjustment for system-related and frailty confounders.

Our findings align with previous research suggesting that early mobilization is associated with improved overall outcomes for patients recovering from hip fractures surgery [11–16]. However, little attention has been given to postoperative pain and opioid use. A study on 52 hip fracture patients in a hospital in Turkey found association between early mobilization and reduced pain level 4 weeks after surgery [29]. However, a study based on 284 hip fracture patients from China reported that neither early or late mobilization group returned to their preinjury levels of EQ-5D VAS pain and discomfort items, and that there was no difference between groups in pain score at 12-week postoperative [30]. Likewise, a study based on 219 hip fracture patients treated at single hospital in Switzerland found no association between early mobilization and pain at discharge after median length of hospital stay of 9.6 days [31]. Studies were limited by low sample size and lack of comparison methods adjusting for potential confounding.

Several mechanisms may explain our finding of the association between early mobilization and reduced postoperative opioid use. Early mobilization plays a role in counteracting the adverse physiological effects of immobilization, such as loss of muscle strength and endurance, soft tissue and bone deterioration, increased risk of thromboembolic events, and impaired function of pulmonary and gastrointestinal systems, inevitably leading to complication [32, 33]. Postoperative complications can result in prolonged bed rest and additional pain, potentially requiring excessive opioid use for sufficient pain relief [11, 34]. Early mobilization improves the clinical outcome of hip fracture patients by reducing rates of painful complications [16]. Patients who begin mobilization early often regain their functional ability more quickly, leading to increased independence and reduced reliance on opioids [35]. Early mobilization is associated with better mental health in terms of reduced risk of depression and anxiety, which can lead to lower opioid use [36].

Reducing the risk of long-term opioid therapy is important due to its association with adverse side effects, such as constipation, physical dependence, tolerance, respiratory depression, osteoporosis, and increased risk of falls and fractures [9, 37]. Our sIPT weighted and subgroup analyses revealed a gradient, where earlier mobilization times—particularly within the first 12 h—were associated with the lowest rates of long-term opioid therapy. This suggests that clinical strategies should not only prioritize mobilization within 24 h but actively aim to facilitate even earlier mobilization when feasible, as additional benefits may be gained by intervening in this narrower window. Further benefits may be seen from ongoing support for mobilization coupled with patient education regarding mobilization, pain, and safe opioid use [38, 39]. Indeed, pain rehabilitation programs that educate patients and their families about the adverse side effects of opioids have been shown to improve physical functioning, mood, and pain severity among patients with chronic pain, hereby decreasing opioid usage [40].

The implementation of enhanced recovery after surgery programs, including multimodal opioid-sparing techniques and early mobilization has shown a reduction in opioid prescription fills up to 180 days after hip fracture surgery [41]. Ideal analgesic strategies are still debated [42] but include multimodal pain relief techniques, such as nonsteroidal anti-inflammatory drugs, selective cyclooxygenase-II inhibitors, local infiltration analgesia, nerve block [43–45], and the postoperative use of less addictive opioids, such as morphine [46].

The risk reduction of 3.44% achieved by early mobilization might seem small with questionable clinical significance, but it should be weighed against the frequency and seriousness of adverse effects of opioids among fragile and multimorbid hip fracture patients [47].

A significant strength of this study lies in using nationwide registries with high validity. The positive predictive values for hip fracture diagnoses and operative procedures are over 90% in these databases [20]. To address potential confounding, we applied the sIPT weighting method, which effectively balanced confounders between groups [48] supporting the validity of our weighted comparisons.

Despite these strengths, there are some limitations to consider.

Smaller group sizes, particularly in the 24–36 h and > 36 h subgroups, may result in less precise estimates with wider confidence intervals, potentially reducing the ability to detect statistically significant differences in these subgroups. Nevertheless, even the smallest group included over 1,700 patients, which provides sufficient statistical power for meaningful comparisons. While a more even distribution might have improved the precision of subgroup estimates, the large cohort size and our statistical approach minimize the influence of this limitation.

In addition, we lacked data regarding clinical indications for prescribing opioids to differentiate between need for opioids due to hip fracture surgery or due to other known conditions. Compliance with opioids is unknown which could result in overestimation of opioid use. However, a study by Schneeweiss & Avorn [49] found that electronic prescription databases still provide a reliable measure of medication intake. Furthermore, while not available in this data set, a comparison of pain scores and opioid use at different timepoints (acute/long-term), as well as a more detailed breakdown of surgery subtypes within the osteosynthesis group, would have provided valuable insights into early mobilization status and opioid consumption patterns.

We had 12% of patients with missing data on the time of mobilization. The true estimate of association of early mobilization and opioid use could be either over- or underestimated, depending on the true mobilization status of the patients with missing data.

Finally, while we used sIPT to account for measured confounding, we cannot entirely exclude the possibility of unmeasured confounding or reverse association between health status, early mobilization and long-term opioid therapy.

## Conclusion

Long-term opioid therapy is a common complication after hip fracture surgery. Mobilization within 24 h after surgery is associated with a lower risk of long-term opioid therapy compared to mobilization between 24 and 36 h. Early mobilization is one of the key elements of the successful patient recovery and reduced rate of complications and mortality after hip fracture surgery.

## Supplementary Information

Below is the link to the electronic supplementary material.

Supplementary file1 (DOCX 14 KB)

## Data Availability

To protect the privacy of patients, it is by Danish law prohibited to make individual level data publicly available.
