# Supplementary material for: Association between early mobilization after hip fracture surgery and risk of long-term opioid therapy
Source: Eur Geriatr Med. 2025 May 7;16(3):921–31. doi: 10.1007/s41999-025-01227-7 (PMC12174293; doi:10.1007/s41999-025-01227-7)
Supplement: Supplementary file 1 — Supplementary file1 (DOCX 14 KB) [file 41999_2025_1227_MOESM1_ESM.docx]

**Supplementary information**

**SI Table 1.** Diagnosis, surgery and ATC codes used to define the study population and long-term opioid therapy

| **Study population** | |
| --- | --- |
|  | **ICD-10 and NOMESCO surgery codes** |
| **Hip fracture diagnosis** | |
| Collum femoris fracture | DS72.0 |
| Pertrochantric femur fracture | DS72.1 |
| Subtrochanteric femur fracture | DS72.2 |
| **Hip fracture surgery** | |
| Hip arthroplasty | All KNFB codes:  KNFB0, KNFB1, KNFB20, KNFB30, KNFB40, KNFB59, KNFB99, |
| Osteosynthesis | KNFJ4, KNFJ5, KNFJ6, KNFJ7, KNFJ8, KNFJ9 |
|  | **Statistics Denmark codes** |
|  | **ATC codes** |
| **Opioids** | |
| Buprenorphine | N02AE01 |
| Codeine & paracetamol | N02AJ06 |
| Fentanyl | N02AB03 |
| Hydromorphone | N02AA03 |
| Ketobemidone & antispasmodics | N02AG02 |
| Methadone | N07BC02 |
| Morphine | N02AA01 |
| Nicomorphine | N02AA04 |
| Oxycodone | N02AA05 |
| Oxycodone + naloxone | N02AA55 |
| Pethidine | N02AB02 |
| Tapentadol | N02AX06 |
| Tramadol | N02AX02 |

**Abbreviations:** ATC, Anatomical Therapeutic Chemical Classification-codes; ICD-10, the International Classification of Diseases, Tenth Revision; NOMESCO, The Nordic Medico-Statistical Committee
